# Veterinary Medical Association of New York County

**Published:** 1896-07

**Authors:** Robert W. Ellis

**Affiliations:** Secretary


### VETERINARY MEDICAL ASSOCIATION OF NEW YORK COUNTY.

The regular meeting of the Association was called to order Tuesday, June 2d, at 8.30 P.M., at the Academy of Medicine, by the President, Dr. Huidekoper. On roll-call the following members responded, viz.: Drs. Bretherton, J. S. Cattanach, Delaney, Ellis, Glover, Huidekoper, Hanson, Lellman, MacKellar, Neher, and O'Shea. The minutes of the previous meeting were read and approved.

Reports of committees: Judiciary Committee, Dr. O'Shea, Chairman, reported the passage of the Cole Bill, and made a brief report as to what the committee was doing with non-registered men. Moved and seconded that the report be accepted; carried.

Committee on Alteration in By-laws, Dr. Hanson, Chairman, reported that the committee offered the following alteration in Article XIII. of the By-laws, to read: "The Association shall meet upon the first Wednesday of each month at the Academy of Medicine, except during July, August, and September," instead of "The Association shall meet upon the first Tuesday of each month, except during July, August, and September." Moved and seconded that the report be accepted; carried.

Moved and seconded that registered veterinary graduates of Kings, Queens, Richmond, and Westchester Counties be eligible to membership in the Veterinary Medical Association of New York County; carried.

Moved and seconded that the Secretary invite practitioners from said counties to the October meeting; carried.

Moved and seconded that the meeting adjourn; carried.

ROBERT W. ELLIS, D.V.S.,
Secretary.

